# Changing epidemiology of malaria in Sabah, Malaysia: increasing incidence of *Plasmodium knowlesi*

**DOI:** 10.1186/1475-2875-13-390

**Published:** 2014-10-02

**Authors:** Timothy William, Jenarun Jelip, Jayaram Menon, Fread Anderios, Rashidah Mohammad, Tajul A Awang Mohammad, Matthew J Grigg, Tsin W Yeo, Nicholas M Anstey, Bridget E Barber

**Affiliations:** Infectious Diseases Unit, Clinical Research Centre, Queen Elizabeth Hospital, Kota Kinabalu, 88560 Sabah Malaysia; Infectious Diseases Society Sabah-Menzies School of Health Research Clinical Research Unit, Kota Kinabalu, 88560 Sabah Malaysia; Sabah Department of Health, Kota Kinabalu, 88814 Sabah Malaysia; Department of Medicine, Clinical Research Centre, Queen Elizabeth Hospital, Kota Kinabalu, 88560 Sabah Malaysia; Sabah State Public Health Laboratory, Kota Kinabalu, 88850 Sabah Malaysia; Menzies School of Health Research, PO Box 41096, Casuarina, NT 0811 Australia; Lee Kong Chian School of Medicine, Nanyang Technological University, Singapore, 308232 Singapore; Department of Infectious Diseases, Royal Darwin Hospital, Darwin, 0811 Northern Territory Australia

**Keywords:** *Plasmodium knowlesi*, Malaria, Epidemiology

## Abstract

**Background:**

While Malaysia has had great success in controlling *Plasmodium falciparum* and *Plasmodium vivax*, notifications of *Plasmodium malariae* and the microscopically near-identical *Plasmodium knowlesi* increased substantially over the past decade. However, whether this represents microscopic misdiagnosis or increased recognition of *P. knowlesi* has remained uncertain.

**Methods:**

To describe the changing epidemiology of malaria in Sabah, in particular the increasing incidence of *P. knowlesi*, a retrospective descriptive study was undertaken involving a review of Department of Health malaria notification data from 2012–2013, extending a previous review of these data from 1992–2011. In addition, malaria PCR and microscopy data from the State Public Health Laboratory were reviewed to estimate the accuracy of the microscopy-based notification data.

**Results:**

Notifications of *P. malariae/P. knowlesi* increased from 703 in 2011 to 815 in 2012 and 996 in 2013. Notifications of *P. vivax* and *P. falciparum* decreased from 605 and 628, respectively, in 2011, to 297 and 263 in 2013. In 2013, *P. malariae/P. knowlesi* accounted for 62% of all malaria notifications compared to 35% in 2011. Among 1,082 *P. malariae/P. knowlesi* blood slides referred for PCR testing during 2011–2013, there were 924 (85%) *P. knowlesi* mono-infections, 30 (2.8%) *P. falciparum*, 43 (4.0%) *P. vivax*, seven (0.6%) *P. malariae*, six (0.6%) mixed infections, 31 (2.9%) positive only for *Plasmodium* genus, and 41 (3.8%) *Plasmodium*-negative. *Plasmodium knowlesi* mono-infection accounted for 32/156 (21%) and 33/87 (38%) blood slides diagnosed by microscopy as *P. falciparum* and *P. vivax,* respectively. Twenty-six malaria deaths were reported during 2010–2013, including 12 with ‘*P. malariae/P. knowlesi*’ (all adults), 12 with *P. falciparum* (seven adults), and two adults with *P. vivax*.

**Conclusions:**

Notifications of *P. malariae/P. knowlesi* in Sabah are increasing, with this trend likely reflecting a true increase in incidence of *P. knowlesi* and presenting a major threat to malaria control and elimination in Malaysia. With the decline of *P. falciparum* and *P. vivax*, control programmes need to incorporate measures to protect against *P. knowlesi*, with further research required to determine effective interventions.

**Electronic supplementary material:**

The online version of this article (doi:10.1186/1475-2875-13-390) contains supplementary material, which is available to authorized users.

## Background

Malaysia has achieved great success in controlling malaria over recent decades, with marked reductions in the incidence of *Plasmodium falciparum* and *Plasmodium vivax*, and a goal of eliminating these species by 2020 [1, 2]. However, there has been an apparent recent increase in the incidence of malaria from the simian parasite *Plasmodium knowlesi,* with combined notifications of *P. knowlesi* and the microscopically near-identical *Plasmodium malariae* increasing more than ten-fold in the north-eastern Malaysian state of Sabah between 2004 and 2011 [3]. Given the rarity of PCR-confirmed *P. malariae* in Sabah [4–6], this increase in notifications is presumed due to an increase in cases of *P. knowlesi* [3]. However, the possibility that this apparent increase is due to increased recognition of the species has not yet been discounted, with clinicians and microscopists undoubtedly more aware of *P. knowlesi* following the first report of human cases of knowlesi malaria in Sarawak in 2004 [7] and subsequent reports of cases widespread across Malaysia [8, 9]. Microscopic diagnosis of *P. knowlesi* is known to be problematic [10], and hence the *Plasmodium* species distribution among *P. malariae/P. knowlesi* microscopy-based notifications remains uncertain.

*Plasmodium knowlesi* has a 24-hour replication cycle and can result in a high parasitaemia with consequent complications [8]. Risk of severe disease in adults appears higher than that of falciparum malaria [11], and fatal cases have been reported [8, 12–15]. Given the potential for this species to be transmitted from human to human [16], and the public health implications of this zoonosis becoming established within human populations, ongoing monitoring and reporting of *P. knowlesi* in Malaysia is crucial to guide further research and the development of malaria control programmes.

This retrospective descriptive study involved a review of Sabah Department of Health malaria notification data from 2012–2013 and extends a previous review of these data from 1992–2011 [3]. In addition, PCR and microscopy results from the State Public Health Laboratory for the years 2010–2013 were obtained. The study aimed to use the Sabah malaria notification database in addition to the State Public Health Laboratory PCR and microscopy results to clarify whether the true incidence of *P. knowlesi* is increasing. In addition, epidemiological features of knowlesi malaria in Sabah were assessed, including age, sex and geographic distribution.

## Methods

### Ethics statement

The study was approved by the Medical Review and Ethics Committee of the Ministry of Health, Malaysia, and the Human Research Ethics Committee of Menzies School of Health Research, Australia.

### Review of malaria notification data

In Sabah, notification of all malaria cases to the State Health Department is mandatory, with notifications based on microscopy results. Blood slides with parasites resembling *P. knowlesi* or *P. malariae* are reported, and hence notified, as *P. malariae*, *P. knowlesi*, or *P. malariae/P. knowlesi*. For analysis purposes these notifications were considered a single group and are referred to as ‘*P. malariae/P. knowlesi*’*.*

Sabah Department of Health malaria notification records from 1992–2011 have been previously reviewed [3]. For the current study Sabah malaria notification records from 2012 and 2013 were reviewed, with data from 2004–2011 also included in this report for comparison purposes. Demographic and epidemiological information for individual notifications was available from 2007.

### Review of malaria PCR and microscopy data from the State Public Health Laboratory

PCR results of all malaria samples referred to the State Public Health Laboratory from January 2010-December 2013 were obtained. Age, sex and microscopy results were included in the database, with this information obtained from the PCR request forms. For the comparison of microscopy and PCR results, data from July 2011-December 2013 were included, and only samples with a PCR request form stating a species-specific microscopic diagnosis (67% of samples) were included in the analysis. The commencement of the specified time period was chosen to coincide with the introduction at the State Public Health Laboratory of a real-time PCR assay for the detection of *P. knowlesi* [17], that replaced a nested PCR assay that has been reported to cross-react with *P. vivax* DNA [18] and resulted in a likely overdiagnosis of *P. knowlesi/P. vivax* mixed infections [5]. *Plasmodium falciparum*, *P. vivax*, *P. malariae* and *Plasmodium ovale* were detected using a multiplexed real-time PCR assay as previously described [19].

Sabah State health policy currently requires all samples diagnosed by microscopy as *P. malariae/P. knowlesi* to be referred to the State Public Health Laboratory for PCR testing. In addition, laboratories are requested to refer approximately 10-15% of randomly selected *P. falciparum* and *P. vivax* blood slides for PCR testing for quality control. The results reported here do not therefore reflect the malaria species distribution in Sabah.

### Rainfall data

To assess whether rainfall may have influenced malaria trends in Sabah, monthly rainfall data recorded at Sabah’s six meteorology stations (Kudat, Keningau, Ranau, Tawau, Sandakan, and Kota Kinabalu) were obtained from the Malaysian Department of Meteorology. Rainfall data were obtained from January 2009 for Kudat, Tawau, Sandakan, and Kota Kinabalu; from August 2009 for Keningau; and from July 2012 for Ranau.

### Data analysis

Data were analysed using Stata statistical software, version 10.0. Median ages were compared using Wilcoxon rank-sum test, and proportions were assessed using the Chi-squared test. For the calculation of *P. knowlesi* incidence rates, district populations were calculated using the 2010 Malaysian Census [20], and the Sabah Department of Health estimates of population growth from 2010–2012 [21]. Spearman’s correlation coefficient was used to assess the association between rainfall and monthly *P. knowlesi/P. malariae* notifications, with cross-correlations analysed to determine the time lag at which the strongest association occurred. Edward’s test was used to assess seasonality.

## Results

### Malaria notification trends in Sabah

**Figure 1:**
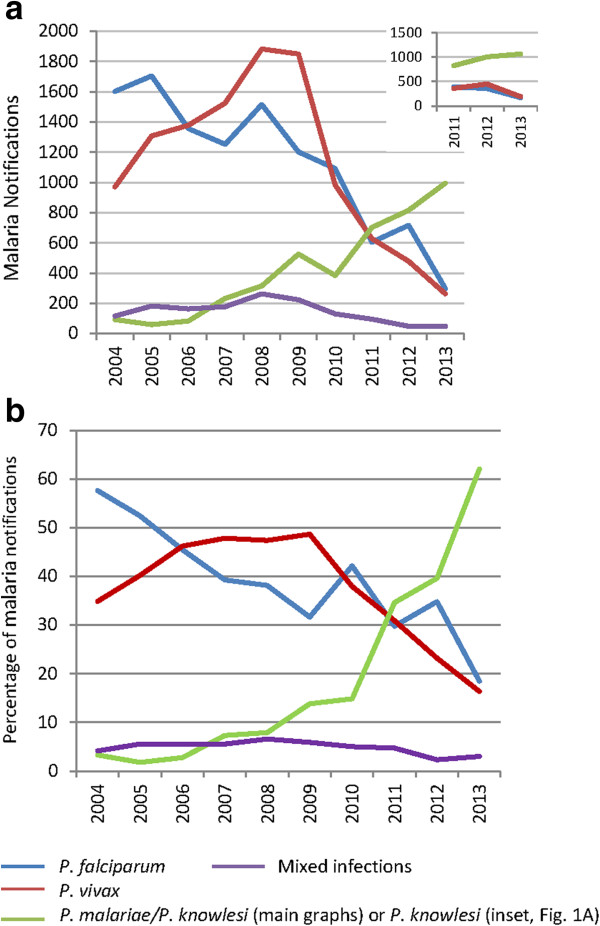
**Malaria notifications by species, 2004–2013. a**. Inset graph shows malaria notifications 2011–2013 adjusted according to the estimated over/under diagnosis of each species calculated from the available microscopy and corresponding PCR results obtained from the State Reference Laboratory (data/calculations shown in Additional file 1). **b**. Percentage of total malaria notifications, 2004–2013.

As previously reported, notifications of ‘*P. malariae/P. knowlesi*’ in Sabah increased markedly from around the mid-2000s, increasing > ten-fold between 2004 (n = 59) and 2011 (n = 703). This increase in notifications has continued, with 815 and 996 cases of *P. malariae/P. knowlesi* notified in 2012 and 2013, respectively (Figure 1a). The decrease in notifications of *P. falciparum* and *P. vivax* has also continued, with cases falling from 605 and 628, respectively, in 2011, to 297 and 263, respectively, in 2013. Consequently, *P. malariae/P. knowlesi* notifications now comprise the large majority of malaria notifications in Sabah, accounting for 62% of all malaria notifications in 2013 compared to 35% in 2011 (Figure 1b).

### Microscopy and PCR results from State Public Health Laboratory

From July 2011 to December 2013 a total of 1,366 samples were referred for *Plasmodium* PCR testing, and had an accompanying request form stating a microscopic diagnosis. Among 1,082 samples diagnosed as *P. malariae/P. knowlesi*, *P. knowlesi* mono-infection was detected in 924 (85%; Table 1). Thirty (2.8%) and 43 (4.0%) were found to be *P. falciparum* and *P. vivax* mono-infections, respectively, while only seven (0.6%) were *P. malariae* mono-infection and six (0.6%) were mixed infections. In contrast, among samples diagnosed by microscopy as *P. falciparum* and *P. vivax* mono-infection, 32/156 (21%) and 33/87 (38%), respectively, were *P. knowlesi* mono-infection by PCR. As might be expected with an increasing predominance of *P. knowlesi* infections in Sabah, the proportion of PCR-confirmed *P. knowlesi* mono-infections among cases diagnosed by microscopy as *P. malariae/P. knowlesi* increased from 2011 to 2013, as did the proportion of *P. knowlesi* mono-infections among microscopy-diagnosed *P. falciparum* infections (Table 2).

**Table 1:** **PCR results among microscopy-diagnosed** Plasmodium malariae/ Plasmodium knowlesi* **,** ***Plasmodium falciparum*** **and** ***Plasmodium vivax*** **mono-infections, and mixed species infections, July 2011-December 2013**

	Microscopy result				
PCR result	Pm/Pk*	Pf	Pv	Mixed infections	Total
**Pk**	924 (85)	32 (21)	33 (38)	23 (56)	1012
**Pf**	30 (2.8)	101 (65)	2 (2.3)	3 (7.3)	136
**Pv**	43 (4.0)	4 (2.6)	42 (48)	7 (17)	96
**Pm**	7 (0.6)	0 (0)	0 (0)	0 (0)	7
**Pk/Pv**	2 (0.2)	1 (0.1)	0 (0)	0 (0)	3
**Pk/Pf**	3 (0.3)	0 (0)	0 (0)	0 (0)	3
**Pf/Pv**	0 (0)	0 (0)	2 (2.3)	0 (0)	2
**Pf/Pm**	1 (0.1)	0 (0)	0 (0)	0 (0)	1
**P. genus** ^**#**^	31 (2.9)	9 (5.8)	3 (3.4)	6 (15)	49
**Negative**	41 (3.8)	9 (5.8)	5 (5.7)	2 (4.9)	57
**Total**	1082	156	87	41	1366

Numbers are N (%). Microscopy results were obtained from data provided on the PCR request form. Only PCR request forms that stated a species-specific microscopy result (67% of request forms) were included in this analysis. Pk = *P. knowlesi*, Pf = *P. falciparum*, Pv = *P. vivax*, Pm = *P. malariae.*

*Microscopic diagnoses of *P. knowlesi* and *P. malariae* were considered as a single group.

^#^Samples found to be *Plasmodium*-positive by PCR but negative in the species-specific PCR assays.

**Table 2:** **Proportion of PCR-confirmed** ***Plasmodium knowlesi*** **mono-infections among microscopy-diagnosed** ***Plasmodium malariae/ Plasmodium knowle*** **s** ***i*** *, ***Plasmodium falciparum*** **and** ***Plasmodium vivax*** **mono-infections, by year**

	Proportion (%) of PCR-confirmed ***P. knowlesi***monoinfections			
Microscopy diagnosis	2011 ^#^	2012	2013	P value
*P. malariae* or *P. knowlesi*	129/163 (79)	327/383 (85)	468/536 (87)	0.035
*P. falciparum*	7/47 (15)	10/65 (15)	15/44 (34)	0.022
*P. vivax*	5/17 (29)	13/30 (43)	15/40 (38)	0.638

*Microscopic diagnoses of *P. knowlesi* and *P. malariae* were considered as a single group.

^#^Data used from July 2011.

In order to estimate the effect that microscopic misdiagnosis may have had on the Sabah malaria notification data, for the years 2011–2013 available State Public Health Laboratory microscopy and corresponding PCR data were used to calculate ‘adjusted notification rates’ of each species (Additional file 1; Figure 1a, inset). Based on these calculations, true *P. knowlesi* notifications were estimated to have increased from 828 in 2011 to 1,067 in 2013 (Additional file 1, Figure 1a, inset).

From July 2011-December 2013 there were a total of 17 PCR-confirmed *P. malariae* cases (including seven diagnosed by microscopy as *P. malariae/P. knowlesi*, and ten without a microscopic diagnosis stated). Relevant travel history was recorded in the Department of Health database for 12 of these cases, with 11 documented as locally acquired and one imported from Africa.

### Sabah malaria notification trends and incidence by district, 2011–2013

**Figure 2:**
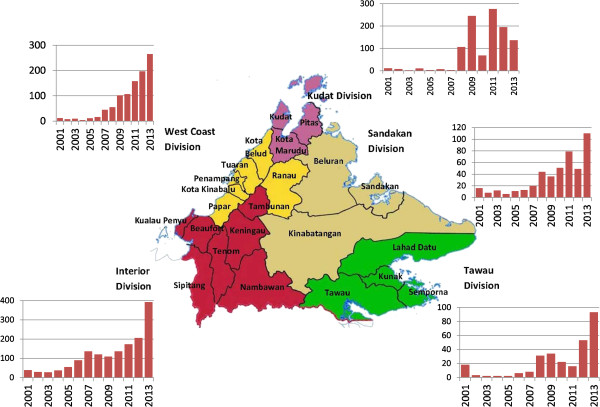
**Map showing districts and division of Sabah, with bar graphs showing annual** ***Plasmodium malariae*** **/** ***Plasmodium knowlesi*** **notifications, by division, from 2001–2013.**

**Figure 3:**
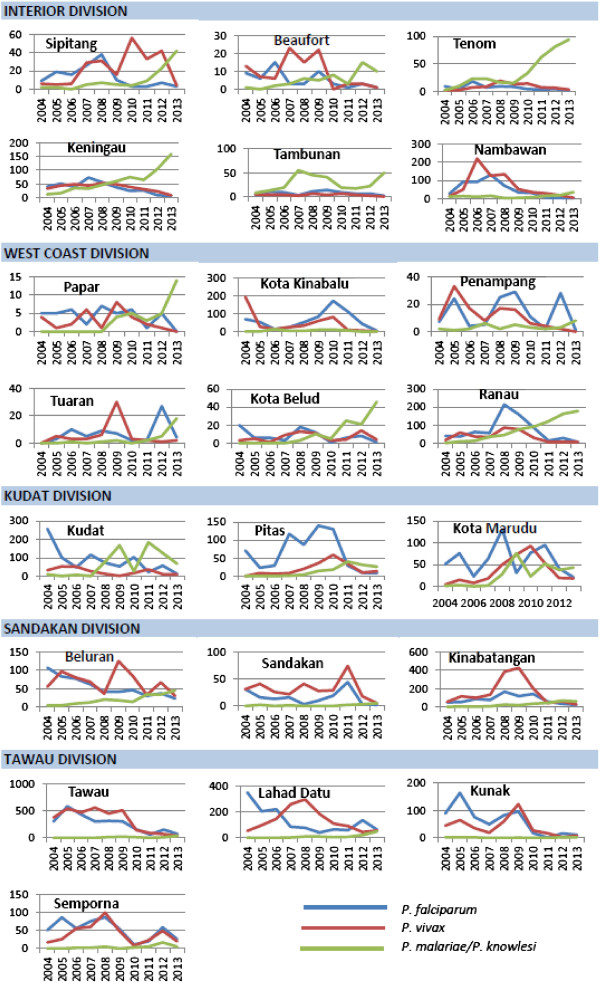
**Malaria notifications by species and district, 2004–2013.**

**Figure 4:**
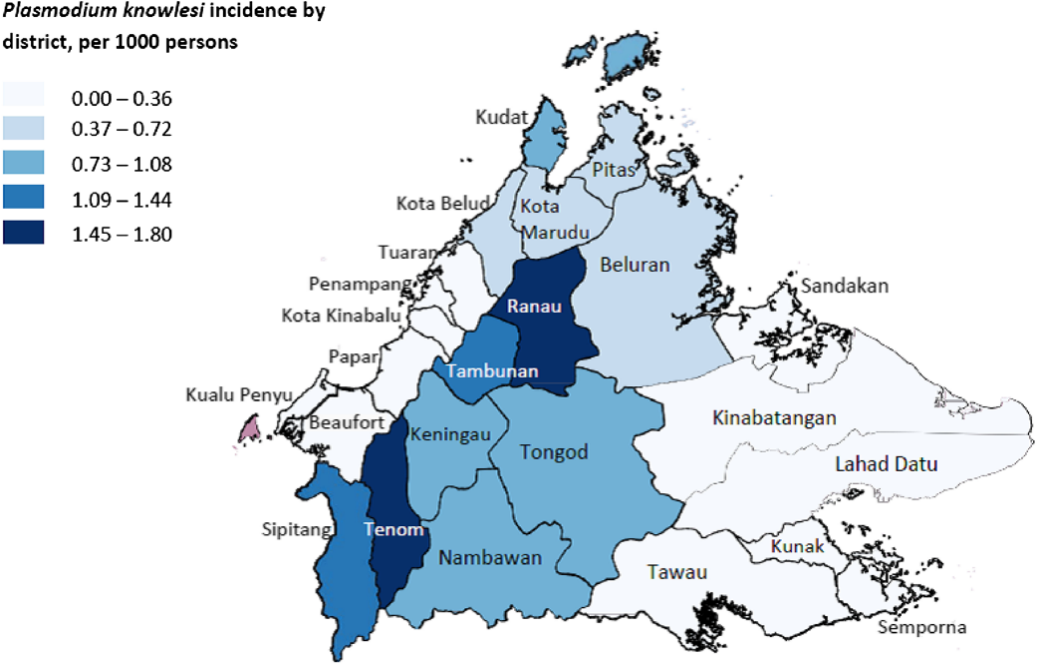
***Plasmodium*** **incidence by district, per 1,000 persons.**

Sixteen of 23 districts in Sabah have experienced a continued increase in notifications of *P. malariae/P. knowlesi* (Figures 2 and 3). In the past two years this increase has been particularly marked in the districts located along the Crocker Range, including Sipitang, Tenom, Keningau, Tambunan, and Ranau (Figures 3 and 4). In these five districts alone notifications of *P. malariae/P. knowlesi* have nearly doubled from 274 in 2011 to 523 in 2013, with these districts now accounting for 53% of all *P. malariae/P. knowlesi* notifications in Sabah despite comprising only 12.5% of Sabah’s population.

In 2013 Ranau had the highest incidence of *P. malariae/P. knowlesi* notifications, with an incidence of 1.8 cases per 1,000 persons (Figure 4). Tenom, Tambunan and Sipitang had incidence rates of 1.15-1.61 per 1,000 persons, while Keningau and neighbouring Nambawan and Tongod (previously part of Kinabatangan district) had incidence rates of 0.87-1.08 per 1,000 persons. In contrast the majority of low-lying coastal districts, in particular those of the West Coast Division and Tawau Division, had incidence rates of <0.2 per 1,000 persons.

Kudat Division in the northeast was the only division to experience a decline in notifications of *P. malariae/P. knowlesi* over the past two years. While notifications increased from 3–11 per year during 2001–2007 to 276 in 2011, notifications fell to 195 in 2012 and 136 in 2013.

### Age and sex distribution

**Figure 5:**
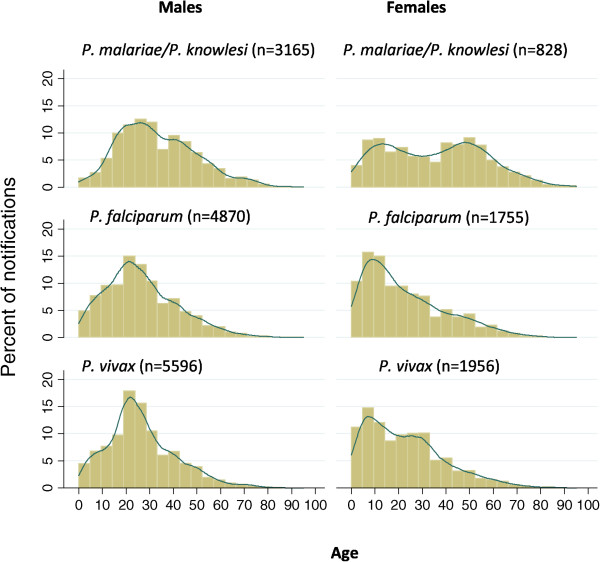
**Age and sex distribution of** ***Plasmodium malariae/Plasmodium knowlesi*** **,** ***Plasmodium falciparum*** **and** ***Plasmodium vivax*** **mono-infections, from 2007–2013.**

From 2007–2013, the overall median age of patients notified with *P. malariae/P. knowlesi* in Sabah (n = 4,015) was 31 years, compared to 23 for those with *P. falciparum* (n = 6,667) and 24 for those with *P. vivax* (n = 7,608, p = 0.0001; Figure 5). For patients notified with *P. malariae/P. knowlesi*, the median age was 31 in the years 2007–2011 compared to 32 in the years 2012–2013 (p = 0.003), while for patients with *P. falciparum* and *P. vivax*, the median age increased from 23 years for both species for the years 2007–2011, to 25 and 27 years, respectively, in 2012, and 29 and 28 years, respectively, in 2013 (p < 0.0001 for both species, for difference in median age between 2007–2011 and 2012–2013). In keeping with the increase in the proportion of *P. malariae/P. knowlesi*, the overall median age of all malaria notifications increased from 24 years during 2007–2010, to 27, 28 and 31 years in the years 2011, 2012 and 2013, respectively (p < 0.0001).

Using PCR results from 2010–2013, the median age of patients with *P. knowlesi* (n = 1,871) was 35 years (IQR 22–49) compared to 27 years (IQR 17–40) for patients with *P. falciparum* (n = 410) and 22.5 years (IQR 13–35) for patients with *P. vivax* (n = 250), with no increase in median age between 2010 and 2013.

Among children < five years old, during 2012–2013 *P. malariae/P. knowlesi* accounted for 19% of all malaria notifications, with 51 and 28% of notifications being *P. falciparum* and *P. vivax,* respectively (with the remainder being mixed infections). During the same time period, *P. malariae/P. knowlesi*, *P. falciparum* and *P. vivax* accounted for 29, 41 and 26%, respectively, of malaria notifications among children aged five to 14 years, and 53, 25 and 19% of malaria notifications among adults ≥15 years.

Among *P. malariae/P. knowlesi* notifications, females were older than males, with a median age of 36 years (IQR 16–52) compared to 31 years (IQR 21–44 years; p = 0.04). This difference was particularly marked among adults ≥15 years, with a median age of 43 years among females and 31 years among males (p < 0.0001). Among adults ≥15 years, males accounted for 82% of all *P. malariae/P. knowlesi* notifications, compared to only 63% of *P. malariae/P. knowlesi* notifications among children (p < 0.0001). Among females, children accounted for 22% of *P. malariae/P. knowlesi* notifications, while children accounted for only 10% of *P. malariae/P. knowlesi* notifications among males (p < 0.0001).

### Malaria deaths

As previously reported, 14 PCR-confirmed malaria deaths were notified in Sabah during 2010–2011, including six with *P. knowlesi* (all adults), seven with *P. falciparum* (four adults) and one adult with *P. vivax* [14]. During 2012 and 2013 a further 11 PCR-confirmed malaria deaths were notified in Sabah, including five with *P. knowlesi* (all adults), five with *P. falciparum* (three adults), and one adult with *P. vivax*, in addition to one adult with a microscopic diagnosis of ‘*P. malariae*’ but with no PCR performed. Overall, from 2010–2013 this represents a notification-mortality rate of 4.1/1,000 (95% CI 2.1-7.2/1000) for *P. malariae*/*P. knowlesi*, 4.4/1,000 (95% CI 2.3-7.7/1000) for *P. falciparum*, and 0.9/1,000 (95% CI 0.1-3.1/1000) for *P. vivax*. Among adults (age >14 years), the notification-mortality rate was 4.6/1,000 (95% CI 2.4-8.0/1000) for *P. malariae*/*P. knowlesi*, 3.5/1,000 (95% CI 1.4-7.3/1000) for *P. falciparum*, and 1.1/1,000 (95% CI 0.1-4.0/1000) for *P. vivax*.

### Correlation between *Plasmodium malariae/Plasmodium knowlesi*notifications and rainfall

**Figure 6:**
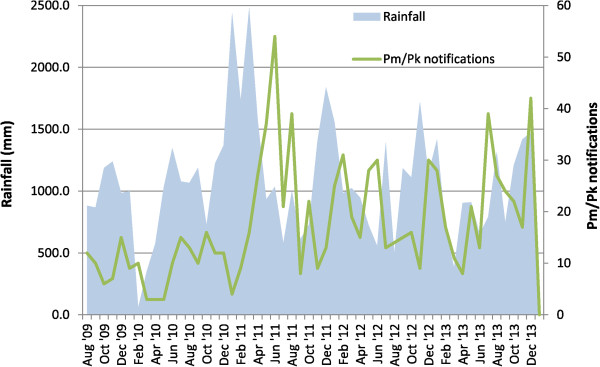
**Monthly rainfall and notifications of** ***Plasmodium malariae/Plasmodium knowlesi*** **, for the districts of Kudat, Keningau, Tawau, Sandakan, and Kota Kinabalu.** For illustrative purposes this figure excludes all data for Ranau as data are only available from July 2012, and all data from Jan-Jul 2009 as rainfall data from Keningau is not available from this time. Calculation of Spearman’s correlation coefficients includes all available data. Spearman’s correlation coefficients for association between monthly rainfall and notifications of *P. malariae/P. knowlesi*: 0.37 (p = 0.004), 0.32 (p = 0.016) and 0.30 (p = 0.024) for months 2, 3 and 4, respectively, following the rainfall.

In the five districts where rainfall data were available, rainfall correlated with notifications of *P. malariae/P. knowlesi* in these districts in the subsequent two to four months, with the correlation peaking at two months (Figure 6). Total rainfall recorded at the five available meteorologic stations fell between 2011 and 2013, with 16,342 mm, 12,815 mm and 11,911 mm recorded in 2011, 2012 and 2013, respectively. Rainfall recorded at the meteorological station in Kudat District fell from 4,221 mm in 2011 to 2,667 mm in 2012 and 1,958 mm in 2013.

**Figure 7:**
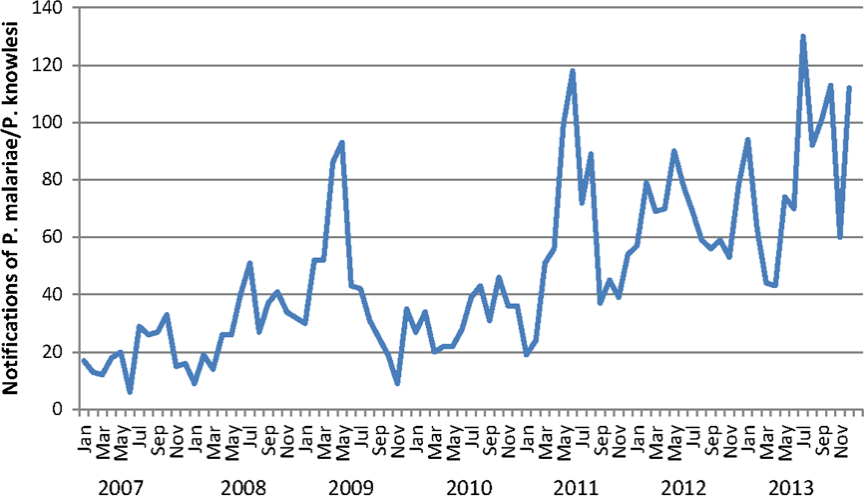
**Monthly notifications of** ***Plasmodium malariae/Plasmodium knowlesi*** **in Sabah.**

Seasonal variation was demonstrated for notifications of *P. malariae/P. knowlesi* from 2007–2013 (p < 0.0001), with notifications peaking during May-August (Figure 7).

## Discussion

This paper demonstrates that notifications of *P. knowlesi*/*P. malariae* in Sabah are continuing to increase, and in 2013 accounted for 62% of all malaria notifications. Furthermore, analysis of microscopy and PCR data from the State Public Health Laboratory demonstrates that this increase in notifications is likely to represent a true increase in incidence of *P. knowlesi* rather than microscopic misdiagnosis of *Plasmodium* species. Although microscopic diagnosis of *Plasmodium* species in Sabah is known to be problematic [10], this study found that over the past three years the microscopic misdiagnosis of true *P. falciparum* or *P. vivax* infections as ‘*P. knowlesi/P. malariae*’ was in fact less common than the misdiagnosis of true *P. knowlesi* as *P. falciparum* or *P. vivax*. The effect of this finding increased from 2011 to 2013, as would be expected with an increasing incidence of *P. knowlesi* and reducing incidence of *P. vivax* and *P. falciparum*, and hence reported malaria notification rates in Sabah may in fact underestimate the predominance of *P. knowlesi* malaria.

The increase in the median age of all malaria notifications, from 24 years during 2007–2011, to 28 years in 2012 and 31 years in 2013, further supports a true increase in the proportion of *P. knowlesi* cases, as patients with PCR-confirmed knowlesi malaria were significantly older than those with PCR-confirmed falciparum or vivax malaria. The increase in median age of patients notified with *P. falciparum* and *P. vivax* is also likely accounted for by a progressive increase in the proportion of these cases actually being *P. knowlesi*.

The increase in notifications of *P. malariae*/*P. knowlesi* has occurred across Sabah, however has been particularly marked in the interior mountainous and more densely forested districts that lie along the Crocker range, which stretches along the southwest-northeast axis of Sabah from Tenom to Ranau. In contrast, incidence has remained relatively low in the more cultivated low-lying districts along the West and East coast. This geographic distribution of knowlesi malaria in Sabah is consistent with forest or forest-edge exposure being a likely risk factor for acquisition of disease; however, further studies are required to confirm the environmental and behavioural risk factors for knowlesi malaria.

Kudat Division was the only division which experienced a decrease in notifications of *P. malariae*/*P. knowlesi* during 2011–2013. Although likely multifactorial, one contributor may have been the decreased rainfall recorded during this time period, with rainfall shown to correlate with notifications of knowlesi malaria.

The malaria trends occurring throughout Sabah have also been observed in the adjacent Malaysian state of Sarawak, where notifications of *P. malariae/P. knowlesi* increased from 685 in 2011 to 737 in 2013 [22]. Notifications of *P. falciparum* fell from 91 in 2011 to 43 in 2013, while notifications of *P. vivax* fell from 935 in 2011 to 216 in 2013. The proportion of *P. malariae/P. knowlesi* notifications as a total of all malaria notifications in Sarawak thus increased from 40% in 2011 to 73% in 2013 [22].

In Peninsular Malaysia, which is geographically separated from Sabah and Sarawak, *P. knowlesi* also accounts for a high proportion of all malaria cases, with a recent study reporting that *P. knowlesi* was detected in 100/218 (46%) microscopy-positive malaria blood samples collected across seven states in Peninsular Malaysia between September 2012 and December 2013 [23]. As in Sabah, the species distribution was found to vary significantly across regions, with *P. knowlesi* detected in 42/56 (75%) and 24/25 (96%) blood samples collected from the eastern Peninsular states of Kelantan and Pahang, respectively. While there are likely differences in the intensity of surveillance in other areas of Southeast Asia where *P. knowlesi* is reported [24], it is notable that the increasing incidence of knowlesi malaria is most marked in Malaysian Borneo and possibly Peninsular Malaysia. While naturally occurring human-to-human transmission has not yet been conclusively demonstrated, its occurrence could account, at least in part, for the observed increase in these regions.

The older age distribution of patients with knowlesi malaria compared to those with falciparum or vivax malaria has been previously reported [3, 5] and is confirmed in this paper. Furthermore, a difference between the age distribution of males and females with knowlesi malaria is confirmed, with females of reproductive age accounting for a smaller proportion of notifications compared to males of this age group. While this may relate to differences in environmental or occupational risk factors among females of this age group, such as lower forest exposure, sex differences in immune response to pathogens are known to occur [25, 26] and may contribute to these findings.

This paper reports that 26 deaths occurred from malaria over the last 4 years, including 11 from PCR-confirmed *P. knowlesi. Plasmodium knowlesi* in adults is associated with high parasitaemia and severity rates at least as high as that of *P. falciparum* [11], and while the case-fatality rate of knowlesi malaria is fortunately low, the ongoing increase in incidence highlights the potential for the absolute number of deaths to increase over coming years. Furthermore, *P. knowlesi* has recently been shown *in vitro* to be capable of adapting to proliferation within human blood, with consequent increase in parasitaemias [27]. Data from the malariotherapy literature indicated that serial passage through humans was associated with increasing virulence [28]. Naturally occurring human-human transmission, if occurring, could result in increasing virulence and associated mortality. Prompt diagnosis and treatment for knowlesi malaria therefore remains paramount, in addition to ongoing monitoring for any changes in the clinical and epidemiological features of disease over time.

The increasing incidence of knowlesi malaria presents a major threat to Malaysia’s goal of eliminating malaria by 2020, and with falciparum and vivax malaria continuing to decline malaria control programmes will need to focus on measures that are effective against *P. knowlesi*. Available evidence suggests that *P. knowlesi* remains primarily a zoonosis, with humans infected when spending time in farms or forested areas in proximity to macaques [29, 30]. A recent study involving mathematical modelling found that long-lasting insecticide-treated nets (LLINs) and hammocks (LLIHs) used in the village and the forest could be expected to reduce human prevalence by 40% [30], and these interventions should be used in high-risk forest or forest-fringe areas. However, it has also been reported that *P. knowlesi* affects all age groups and that familial clusters have occurred, suggesting peridomestic transmission and the possibility of human-human transmission [5]. The use of LLINs in more urban areas may therefore also be beneficial, in addition to rapid treatment of diagnosed cases to prevent onward transmission [30]. Further research is required however to address the substantial knowledge gaps that exist with regards to the transmission dynamics of *P. knowlesi*, including risk factors for acquisition of disease, the identity of the mosquito vector(s) in Sabah, and the extent of human-human transmission.

This study was associated with several limitations. Firstly, malaria notifications in Sabah are based on microscopy results, and hence may not reflect the true *Plasmodium* species distribution. However, available microscopy and corresponding PCR results were used to estimate the overall effect of microscopic misdiagnosis, with ‘adjusted’ notification data supporting the conclusion that the proportion of true *P. knowlesi* cases among all malaria notifications is indeed increasing. Secondly, the use of malaria notification data to estimate malaria incidence trends in Sabah almost certainly underestimates true malaria incidence, given that a substantial number of malaria cases are likely to be unnotified. Furthermore, although notification of malaria cases in Sabah has been mandatory since 1992, the increased recognition of knowlesi malaria over recent years may have changed reporting practices. However, it is unlikely that these factors would have affected the overall species distribution of malaria notifications, and the ongoing and widespread increase in annual notifications of knowlesi malaria nine years after the first report of human *P. knowlesi* infections in Sarawak [7] is further supportive of a true increase in incidence of knowlesi malaria. However, large population-based cross-sectional studies will be required to more accurately describe the true burden and distribution of malaria species in Sabah, while PCR-based longitudinal studies will be required to monitor ongoing trends.

## Conclusions

This paper confirms an ongoing increase in notifications of *P. malariae/P. knowlesi* in Sabah. Analysis of microscopy and PCR data, together with an increase in the median age of all malaria notifications in Sabah, suggests that this trend is likely accounted for by a true increase in incidence of *P. knowlesi* and not by microscopic misdiagnosis or increased recognition of this species. With the decline of *P. falciparum* and *P. vivax* in Sabah, control programmes now need to incorporate measures which will protect against *P. knowlesi*, with further research required to determine effective interventions.

## Electronic supplementary material

Additional file 1: **Calculation of adjusted malaria notifications.** This additional file provides full methods and results for calculation of ‘adjusted malaria notification rates’. These malaria notification rates are adjusted according to the estimated over/under diagnosis of each species calculated from the available microscopy and corresponding PCR results obtained from the State Reference Laboratory. (PDF 384 KB)
