# Supplementary material for: Changing epidemiology of malaria in Sabah, Malaysia: increasing incidence of Plasmodium knowlesi
Source: Malar J. 2014 Oct 2;13:390. doi: 10.1186/1475-2875-13-390 (PMC4195888; doi:10.1186/1475-2875-13-390)
Supplement: Supplementary file 1 — Additional file 1: Calculation of adjusted malaria notifications. This additional file provides full methods and results for calculation of ‘adjusted malaria notification rates’. These malaria notification rates are adjusted according to the estimated over/under diagnosis of each species calculated from the available microscopy and corresponding PCR results obtained from the State Reference Laboratory. (PDF 384 KB) [file 12936_2014_3554_MOESM1_ESM.pdf]

Calculation of adjusted malaria notifications

Methods and Results

In order to estimate the effect that microscopic misdiagnosis may have had on the Sabah malaria notification data (**Supp. Table 1**), for the years 2011 – 2013 we used the available State Public Health Laboratory microscopy and corresponding PCR data to calculate "adjusted notification rates" of each species. To account for the changing relationship between microscopy and PCR results for each species over the years (**Supp. Table 2**), separate calculations were used for each year. For example, in 2011 the total "adjusted notifications" of *P. knowlesi* was calculated as: 85% of total *P. knowlesi/P. malariae* notifications (0.85 x 703) + 15% of *P. falciparum* notifications (0.15 x 605) + 29% of *P. vivax* notifications (0.29 x 628). Complete calculations for all species and for years 2011 - 2013 are shown in **Supp. Table 3**.

Adjusted notifications rates are shown in **Supp. Table 4**, and demonstrate an increase in the adjusted notifications of *P. knowlesi* from 828 in 2011 to 1067 in 2013, with a corresponding decrease in adjusted notifications of *P. falciparum* (388 to 168) and *P. vivax* (360 to 190).

Supplementary Table 1. Actual notifications of *P. malariae/P. knowlesi, P. falciparum* and *P. vivax*, by year

	Year		
**Species**	**2011**	**2012**	**2013**
P. malariae/P. knowlesi	**703**	**815**	**996**
P. falciparum	**605**	**716**	**297**
P. vivax	**628**	**478**	**263**

Supplementary Table 2. Proportion of PCR-confirmed *P. knowlesi, P. falciparum* and *P. vivax* monoinfections among blood slides diagnosed by microscopy as "*P. malariae/P. knowlesi*", *P. falciparum*, and *P. vivax*, by year

	Year		
	**2011***	**2012**	**2013**
**Microscopy diagnosis**	**Proportion (%) of PCR-confirmed *P. knowlesi* monoinfections**		
P. malariae or *P. knowlesi*	129/163 (79)	327/383 (85)	468/536 (87)
P. falciparum	7/47 (15)	10/65 (15)	15/44 (34)
P. vivax	5/17 (29)	13/30 (43)	15/40 (38)
	**Proportion (%) of PCR-confirmed *P. falciparum* monoinfections**		
P. malariae or *P. knowlesi*	3/163 (1.8)	20/383 (5.2)	7/536 (1.3)
P. falciparum	29/47 (62)	50/65 (77)	22/44 (50)
P. vivax	0/17 (0)	1/30 (3.3)	1/40 (2.5)
	**Proportion (%) of PCR-confirmed *P. vivax* monoinfections**		
P. malariae or *P. knowlesi*	9/163 (5.5)	13/383 (3.4)	21/536 (3.9)
P. falciparum	2/47 (4.3)	1/65 (1.5)	1/44 (2.3)
P. vivax	8/17 (47)	12/30 (40)	22/40 (55)

*Data used from July 2011

Supplementary Table 3. Equations used to calculate adjusted notification rates:

**2011**
Adjusted notifications of *P. knowlesi* = (0.79 x 703) + (0.15 x 605) + (0.29 x 628) = 828
Adjusted notifications of *P. falciparum* = (0.018 x 703) + (0.62 x 605) + (0 x 628) = 388
Adjusted notifications of *P. vivax* = (0.055 x 703) + (0.043 x 605) + (0.47 x 628) = 360
**2012:**
Adjusted notifications of *P. knowlesi* = (0.85 x 815) + (0.15 x 716) + (0.43 x 478) = 1006
Adjusted notifications of *P. falciparum* = (0.052 x 815) + (0.77 x 716) + (0.033 x 478) =596
Adjusted notifications of *P. vivax* = (0.034 x 815) + (0.015 x 716) + (0.40 x 478) = 230
**2013:**
Adjusted notifications of *P. knowlesi* = (0.87 x 996) + (0.34 x 297) + (0.38 x 263) = 1067
Adjusted notifications of *P. falciparum* = (0.013 x 996) + (0.50 x 297) + (0.025 x 263) = 168
Adjusted notifications of *P. vivax* = (0.039 x 996) + (0.023 x 297) + (0.55 x 263) = 190

Supplementary Table 4. Notifications of P. *knowlesi/P. malariae, P. falciparum* and *P. vivax*, adjusted according to the PCR distribution of microscopy-diagnosed malaria slides referred to the State Public Health Laboratory. Unadjusted notification rates are shown in parenthesis.

Microscopy diagnosis	2011	2012	2013
P. malariae or *P. knowlesi*	828 (703)	1006 (815)	1067 (996)
P. falciparum	388 (605)	354 (716)	168 (297)
P. vivax	360 (628)	449 (478)	190 (623)
